# Transgluteal CT-Guided Percutaneous Renal Access for Percutaneous Nephrolithotomy in a Pelvic Horseshoe Kidney

**DOI:** 10.1089/cren.2015.29009.rmu

**Published:** 2015-10-01

**Authors:** Ryan J. Mullins, Casey A. Dauw, Michael S. Borofsky, Nadya York, Aashish A. Patel, James E. Lingeman

**Affiliations:** ^1^ Department of Urology, Indiana University School of Medicine, Indianapolis, Indiana.; ^2^ Department of Urology, IU Health Methodist Hospital, Indianapolis, Indiana.; ^3^ Department of Radiology, IU Health Methodist Hospital, Indianapolis, Indiana.

## Abstract

CT-guided percutaneous renal access has been described as a safe and effective access technique in patients with complex anatomy, including ectopic kidney, retrorenal colon, spinal dysraphism, hepatomegaly, and splenomegaly. In comparison to conventional intraoperative fluoroscopic-guided access, CT imaging allows for delineation of surrounding structures that are at risk for injury during percutaneous access. However, previous reports indicate that pelvic kidneys might be inaccessible percutaneously without laparoscopic assistance. Herein, we present a novel transgluteal route to renal access for percutaneous nephrolithotomy (PCNL) in a patient with a pelvic horseshoe kidney and severe spinal deformity.

## Clinical History

**FIG. 1.**
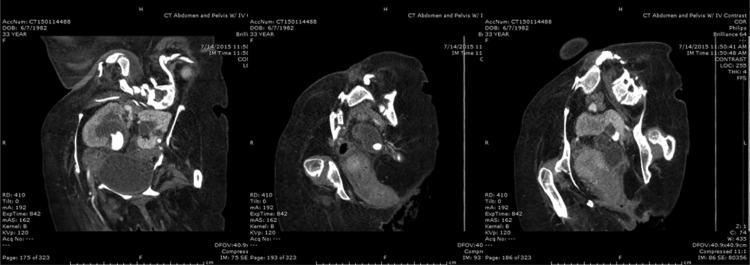
Selected coronal CT images displaying stone burden. From *left* to *right*: right UPJ stone (note curvature of spine); left ureteral stone; left renal stone.

**FIG. 2.**
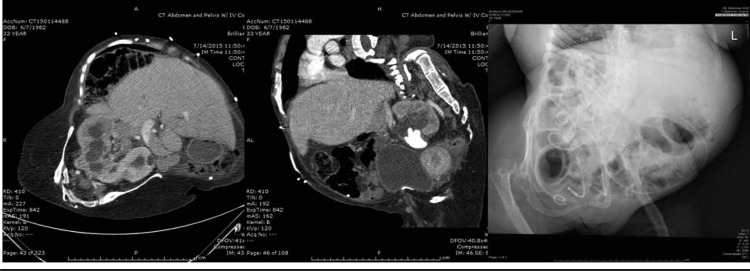
Selected radiographic images displaying complex anatomy. From *left* to *right*: axial CT image with pelvic horseshoe kidney; sagittal CT image showing spinal curvature; radiograph showing skeletal deformities.

J.L. is a 33-year-old female with a medical history notable for myelomeningocele, recurrent *Enterobacter* urinary tract infection (UTI), and no history of urinary calculi, who initially presented to our institution with urosepsis in July 2015. A CT scan (Fig. 1) revealed a pelvic horseshoe kidney with significant bilateral hydronephrosis resulting from a 3.3 cm obstructing stone in the right ureteropelvic junction (UPJ) and several stones in the left renal pelvis and ureter measuring up to 1.5 cm. She underwent bilateral retrograde ureteral stent placement at that time. She received supportive care and was discharged home after a 10-day hospitalization. She subsequently returned to the urology clinic for a discussion of possible treatment options, including bilateral PCNL. Given her complex anatomy (Fig. 2), the patient was also evaluated by radiology for CT-guided percutaneous bilateral renal access to facilitate bilateral PCNL.

## Physical Examination

J.L. was 109.2 cm tall and weighed 38.6 kg. Her BMI was 32.4 kg/m^2^. She had skeletal dysplasia and significant curvature of the spine. She was wheelchair bound and had a stage II sacral decubitus ulcer.

## Diagnosis

Nephrolithiasis with significant bilateral stone burden in a patient with pelvic horseshoe kidney, recurrent UTIs, myelomeningocele, and severe spinal deformity.

## Intervention

**FIG. 3.**
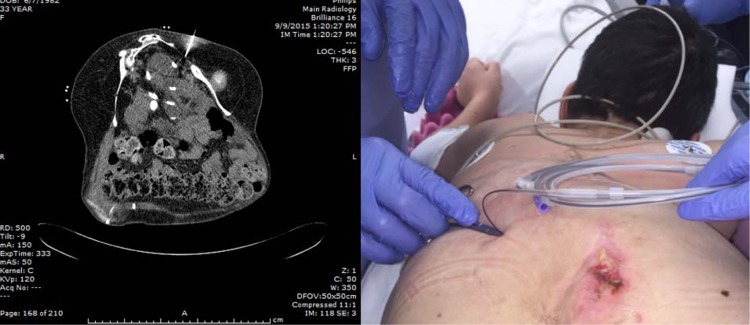
From *left* to *right*: Needle placement through left sciatic foramen using CT guidance; hydrophilic wire entering skin transgluteally to access left renal moiety (note sacral decubitus ulcer).

Before planned bilateral PCNL, J.L. received a 2-week course of meropenem leading up to the day of surgery based on previous antimicrobial sensitivities. On the day of surgery, an interventional radiologist (A.A.P.) obtained bilateral renal access under CT guidance. Access to the right moiety was obtained just superior to the right iliac crest. Due to the more caudal location of the left moiety, a novel approach was used. Access was obtained transgluteally through the greater sciatic foramen (Fig. 3). The urology team was present for the procedure and placed bilateral hydrophilic guide wires to maintain access once needles were confirmed to be in appropriate position.

The patient was then transported to the operating room. In a modified lithotomy position, bilateral ureteral catheters were placed after the previously placed double pigtail ureteral stents were removed. The patient was then transitioned into the prone position. She was secured, prepared, and draped in the usual manner. After making a skin incision on the left side, an angiographic catheter was used to navigate the hydrophilic wire down the ureter. This was then exchanged for the Amplatz Super Stiff™ (Boston Scientific, Marlborough, MA) guidewire. An 8–10French (F) coaxial dilator was then used to place a safety guidewire. The tract was then dilated to 30F using the NephroMax™ (Boston Scientific, Marlborough, MA) balloon, and a sheath was placed. The left renal pelvic stones were fragmented using an ultrasonic lithotripter. Given the dilation of the left ureter, the left ureteral stones could be accessed using a flexible nephroscope and 1.5F nitinol basket. A 10F Cope^®^ (Cook Medical, Bloomington, IN) loop nephrostomy tube and 5F re-entry ureteral catheter were placed. Access to the right side was obtained in the same manner as the left side. Due to difficulty in reaching the stone with the rigid nephroscope, the stone was fragmented using a flexible nephroscope and a 200-μm holmium laser fiber. Fragments were subsequently removed using the ultrasonic lithotripter. Similar to the left side, a 10F Cope^®^ loop nephrostomy tube and 5F reentry ureteral catheter were placed in the right side. Stones from each side were sent for culture and biochemical analysis.

**FIG. 4.**
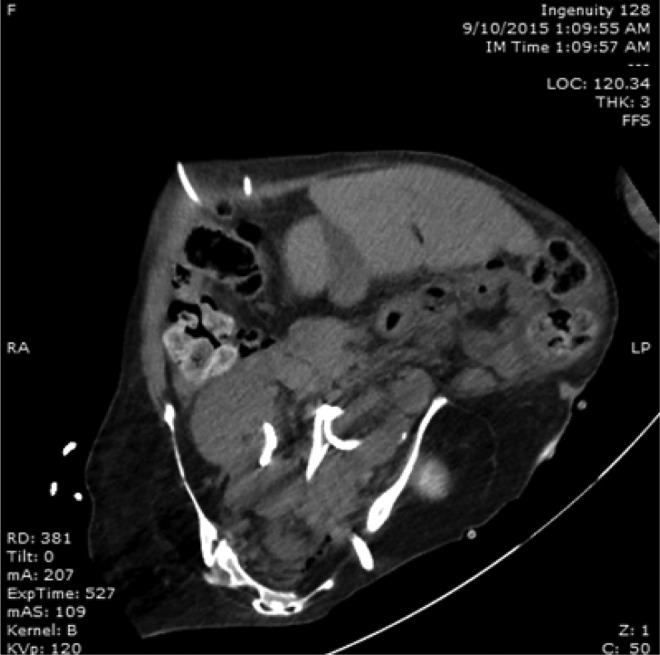
Postoperative CT imaging following primary PCNL. Left nephrostomy tube can be seen entering through the sciatic foramen. A portion of right nephrostomy tube visible in the right renal pelvis.

On postoperative day 1, the patient underwent a CT scan (Fig. 4) that revealed a small amount of residual stone fragments in the left renal pelvis and bladder.

Given concern for infection stones, the patient was taken to the operating room the following day for secondary PCNL. In the lithotomy position, a rigid nephroscope was advanced into the bladder and stone fragments were removed using the ultrasonic lithotripter. The patient was then positioned in the prone position. A guidewire was inserted through the re-entry ureteral catheter. An 8–10F coaxial dilator was then used to place a safety guidewire. A 26F rigid Amplatz renal dilator and sheath were then used to gain access to the calyx of puncture. The remaining stone fragments were removed using a rigid nephroscope and ultrasonic lithotripter. The entire kidney was then carefully inspected using a flexible nephroscope as well as fluoroscopy and was determined to be free of any stones. The patient was discharged later that day with bilateral nephrostomy tubes to dependent drainage. She will complete a 10-day course of ciprofloxacin at home based on previous antimicrobial sensitivities.

Stone culture demonstrated no evidence of infection and biochemical analysis is pending.

## Follow-Up

J.L. was rendered stone free following her secondary PCNL. Bilateral antegrade pyelograms demonstrated good contrast flow to the bladder with no evidence of extravasation.

## Outcomes

J.L. was stone free after her secondary PCNL. She was discharged on hospital day 3 with stable vital signs and laboratory values.

Based on an abundance of literature, PCNL is a safe and effective approach to treat calculi in horseshoe kidneys. Moreover, both complication and stone-free rates approximate those reported for anatomically normal kidneys.^1^ In most cases, percutaneous access can be obtained in a conventional manner using intraoperative fluoroscopic guidance. However, kidneys located in the pelvis are more challenging to access due to surrounding bone structures, complex pelvic vasculature, and adjacent bowel and thus have been reported in the past to require laparoscopic assistance.^2,3^ CT-guided percutaneous renal access has been established as a safe and effective technique in patients with complex anatomy as it allows for delineation of surrounding structures such that they can be avoided.^2,4^ However, pelvic location of the kidney has been reported to be a limitation of direct percutaneous access, even with the use of CT guidance.^2,3^ Although prior reports have demonstrated the feasibility of a transgluteal approach to percutaneous access to the pelvic kidney using fluoroscopy,^5^ this case introduces a novel route to CT-guided percutaneous renal access in a patient with challenging anatomy owing to a pelvic horseshoe kidney and significant spinal deformity.

## Abbreviations Used

CT: computed tomography
PCNL: percutaneous nephrolithotomy
UPJ: ureteropelvic junction
UTI: urinary tract infection

## References

[1] Goswami AK, Shrivastava P, Mukherjee A, Sharma SK Management of colonic perforation during percutaneous nephrolithotomy in horseshoe kidney. J Endourol 2001;15:989–991 11789981 10.1089/089277901317203065

[2] Matlaga BR, Shah OD, Zagoria RJ, Dyer RB, Streem SB, Assimos DG Computerized tomography guided access for percutaneous nephrostolithotomy. J Urol 2003;170:45–47 12796641 10.1097/01.ju.0000065288.83961.e3

[3] Marcovich R, Smith AD Percutaneous renal access: Tips and tricks. BJU Int 2005;95:78–84 15720340 10.1111/j.1464-410X.2005.05205.x

[4] Thanos L, Mylona S, Stroumpouli E, Kalioras V, Pomoni M, Batakis N Percutaneous CT-guided nephrostomy: A safe and quick alternative method in management of obstructive and nonobstructive uropathy. J Endourol 2006;20:486–490 16859461 10.1089/end.2006.20.486

[5] Watterson JD, Cook AJ, Sahajpal R, Bennett J, Denstedt JD Percutaneous nephrolithotomy of a pelvic kidney: a posterior approach through the greater sciatic foramen. J Urol 2001;166:209–210 11435861

